# Integrative Omics Analysis Reveals a Limited Transcriptional Shock After Yeast Interspecies Hybridization

**DOI:** 10.3389/fgene.2020.00404

**Published:** 2020-05-07

**Authors:** Hrant Hovhannisyan, Ester Saus, Ewa Ksiezopolska, Alex J. Hinks Roberts, Edward J. Louis, Toni Gabaldón

**Affiliations:** ^1^Centre for Genomic Regulation, Barcelona Institute of Science and Technology, Barcelona, Spain; ^2^Department of Health and Life Sciences. Universitat Pompeu Fabra, Barcelona, Spain; ^3^Centre for Genetic Architecture of Complex Traits, University of Leicester, Leicester, United Kingdom; ^4^Institució Catalana de Recerca i Estudis Avançats, Barcelona, Spain

**Keywords:** hybridization, yeast hybrid, transcriptome shock, allele-specific expression, buffering

## Abstract

The formation of interspecific hybrids results in the coexistence of two diverged genomes within the same nucleus. It has been hypothesized that negative epistatic interactions and regulatory interferences between the two sub-genomes may elicit a so-called genomic shock involving, among other alterations, broad transcriptional changes. To assess the magnitude of this shock in hybrid yeasts, we investigated the transcriptomic differences between a newly formed *Saccharomyces cerevisiae* × *Saccharomyces uvarum* diploid hybrid and its diploid parentals, which diverged ∼20 mya. RNA sequencing (RNA-Seq) based allele-specific expression (ASE) analysis indicated that gene expression changes in the hybrid genome are limited, with only ∼1–2% of genes significantly altering their expression with respect to a non-hybrid context. In comparison, a thermal shock altered six times more genes. Furthermore, differences in the expression between orthologous genes in the two parental species tended to be diminished for the corresponding homeologous genes in the hybrid. Finally, and consistent with the RNA-Seq results, we show a limited impact of hybridization on chromatin accessibility patterns, as assessed with assay for transposase-accessible chromatin using sequencing (ATAC-Seq). Overall, our results suggest a limited genomic shock in a newly formed yeast hybrid, which may explain the high frequency of successful hybridization in these organisms.

## Introduction

Interspecific hybridization, meaning the mating of two different species to produce viable offspring, has been observed across a wide range of eukaryotic taxa and is considered a major mechanism driving adaptation to new environmental niches (Gladieux et al., 2014; Depotter et al., 2016; Session et al., 2016). Hybridization in animals (Schwenk et al., 2008) and plants (Rieseberg, 1997) has long been recognized, and these organisms have focused the attention of most of the studies on addressing the mechanisms and consequences of hybridization. In contrast, hybridization in microbial eukaryotes has been historically neglected, given the difficulty to detect morphological or physiological differences between species and their hybrids. It was the deep physiological and genetic characterization of the model yeast species *Saccharomyces cerevisiae* that allowed the discovery that several strains, initially classified as independent species, were in fact hybrids (Dujon, 2010). More recently, advances in next-generation sequencing (Goodwin et al., 2016) have facilitated the discovery of hybrids, demonstrating that hybridization is more frequent than previously anticipated, particularly in some microbial groups such as fungi (Albertin and Marullo, 2012). Saccharomycotina yeasts seem particularly prone to hybridization (Morales and Dujon, 2012), and there are numerous examples of yeast hybrid lineages of clinical (Pryszcz et al., 2014, 2015; Schröder et al., 2016; Mixão et al., 2019) or industrial (Le Jeune et al., 2007; Baker et al., 2015; Krogerus et al., 2017) relevance. Furthermore, a hybridization event has been proposed to have led to an ancient whole-genome duplication in the lineage leading to *S. cerevisiae* and related yeasts (Marcet-Houben and Gabaldón, 2015).

An immediate outcome of interspecies hybridization is the coexistence of divergent genetic material within the same nucleus. This has been proposed to lead to a state called “genomic shock” (McClintock, 1984), in which negative epistatic interactions between the two coexisting sub-genomes, including interference between their gene regulatory networks, result in large physiological alterations.

Recent research has studied the effects of this “shock” on different layers of cellular organization, including, among others, the genome (Dutta et al., 2017; Smukowski Heil et al., 2017), the transcriptome (Cox et al., 2014; Hu et al., 2016; Lopez-Maestre et al., 2017), the epigenome (Groszmann et al., 2011; Greaves et al., 2015), and the proteome (Guo et al., 2013; Hu et al., 2017). Specifically, the assessment of transcriptomic changes in hybrids has been used for exploring *cis-* and *trans-*regulation of gene expression (Tirosh et al., 2009; Graze et al., 2012; Li and Fay, 2017; Metzger et al., 2017; Waters et al., 2017). The comparison of gene expression levels in hybrid lineages *versus* their respective parents constitutes a versatile model for assessing gene regulation (Wittkopp et al., 2004). Considering that parental genomes in a hybrid are exposed to the same cellular environment, and thus *trans-*regulatory elements, differences in the gene expression levels within a hybrid can be attributed to *cis-*regulation, while the differences observed between parental organisms are due to a combination of *cis* and *trans* effects (Wittkopp et al., 2004). Using this concept, *cis-* and *trans-*regulatory effects on gene expression have been studied in numerous taxa, including fungi (Thompson and Regev, 2009), flies (McManus et al., 2010), and plants (Guo et al., 2008; Combes et al., 2015). Most transcriptomic studies of fungal hybrids have been performed in that particular context. For instance, Tirosh et al. (2009) investigated the impact of *cis* and *trans* effects on gene expression divergence in closely related *S. cerevisiae* and *Saccharomyces paradoxus* and their interspecific hybrid at four different growth conditions. By performing within-hybrid (*cis* effects) comparisons and subtracting those from between-parent comparisons (*trans* effects), the authors demonstrated that the majority of the regulatory divergence was the result of *cis* effects, attributed to differences in the promoter and regulatory regions that were independent of the environmental condition. On the other hand, *trans* effects were related to the transcription and chromatin regulators and were mostly condition-specific.

Using a similar approach, Metzger et al. (2017) used publicly available RNA sequencing (RNA-Seq) datasets of two *S. cerevisiae* strains and their hybrid (Schaefke et al., 2013) and data of *S. cerevisiae*, *S. paradoxus*, *Saccharomyces mikatae*, and *Saccharomyces bayanus* and their respective hybrids (Schraiber et al., 2013) to assess the dynamics of the regulatory changes throughout long evolutionary distances. They concluded that, as sequence divergence increases, *cis-*regulatory divergence becomes the dominant regulatory mechanism and that both differences in the gene expression and regulatory sequences increase with genetic distance, reaching a plateau for distantly related species.

Another study (Li and Fay, 2017) used *S. cerevisiae × Saccharomyces uvarum* hybrid, resulting from the mating of two thermally divergent species, to investigate the effect of temperature on allele-specific expression (ASE). Using RNA-Seq, the authors assessed the ASE patterns in the hybrids grown at different temperatures and showed that most of the *cis* divergence is temperature-independent, with only a small fraction of the ASE genes influenced by thermal condition. Overall, most previous studies used the transcriptomics of hybrids as a means to investigate *cis* and *trans* effects on gene regulation at various conditions and evolutionary distances, but they did not directly assess the impact of hybridization on gene expression and how this compares with the regulatory impact of other stresses. Given their different focus, these studies do not measure gene expression in matched parental pairs and their hybrids across different conditions, preventing the reanalysis of their data for the purpose of assessing the impact of hybridization and how it compares with environmental effects.

The direct consequences of hybridization on the gene expression profiles of parental species have been mostly studied in plants and animals (McManus et al., 2010; Yoo et al., 2013; Li et al., 2014; Wu et al., 2018; Zhang et al., 2018). Though using different methodologies, all these studies report widespread transcriptomic changes following hybridization, 10–30% of the genes being significantly affected. In this context, fungal studies are more limited. Cox et al. (2014) did address this issue in the natural fungal diploid hybrid (allopolyploid) *Epichloë* Lp1 by comparing its expression patterns with those in its haploid parental species. The authors found that this natural hybrid retained most gene copies of the two parental species and, most importantly, that these genes generally retained the gene expression levels from the parental counterparts. In addition, differences in expression between homeologous genes tended to be lower than the corresponding differences between the orthologous genes in the parental species. Based on these findings, the authors concluded that the transcriptional response to hybridization was largely buffered. However, being based on a natural hybrid, this study does not allow discarding the possibility that the lack of strong differences in the gene expression is due to amelioration through compensatory mutations subsequent to the hybridization. In addition, by comparing a diploid hybrid to haploid parentals, that study could not disentangle the effects of ploidy change from those of hybridization.

We here set out to directly assess the immediate transcriptional impact of hybridization and compare it with the effect of an environmental stress. To this end, we conducted an integrative multi-omics study comparing two distantly related fungal species—*S. cerevisiae* (SC) and *S. uvarum* (SU)—and their newly made hybrid at two thermal conditions. Using RNA-Seq, we assessed the transcriptional differences between orthologous genes in the parental species, between genes in the parental and the hybrid genetic background, and between homeologous genes coexisting in the hybrid. To compare the relative impact of hybridization with an environmental stress, we performed these experiments at two different temperatures, of which one affects the two parental species differently. We further investigated the consequences of hybridization on chromatin states by performing an assay for transposase-accessible chromatin using sequencing (ATAC-Seq) and integrated its results with our RNA-Seq data to obtain mechanistic insights behind the transcriptomic alterations caused by interspecific hybridization.

## Materials and Methods

### Strains

The diploid hybrids of *S. cerevisiae* and *S. uvarum* were generated as follows: genetically tractable isogenic *MATa* and *MAT*α haploids of the North American *S. cerevisiae* strain YPS128, isolated from the bark of an oak tree, were previously generated (Cubillos et al., 2009; Liti et al., 2009) by first isolating a single meiotic spore from the wild-type homothallic strain, resulting in complete homozygosity across the genome except for the *MAT* locus. The *HO* gene was then replaced by a hygromycin resistance cassette, resulting in a diploid heterozygous for *HO*. Haploid spores (*ho:HYG MATa* or *MAT*α) were then isolated from this and *URA3* was replaced in these by the G418 resistance cassette *KANMX*. Similarly, the *S. uvarum* strain UWOPS99-807.1.1, isolated from Argentina, was dealt with in the same way, resulting in isogenic haploids of both mating types (Wimalasena et al., 2014). Diploid hybrids were formed by mating the *MATa S. cerevisiae* to the *MAT*α *S. uvarum* and *vice versa*.

### Experimental Conditions and RNA Extraction

Samples for RNA extraction were collected during the mid-exponential growth phase in rich medium [yeast extract peptone dextrose (YPD)] at two different temperatures: 30°C (normal growing temperature for those species; Salvadó et al., 2011) and 12°C (“cold-shock” condition).

Experiments were performed as follows: first, to delimit the timing of the mid-exponential growth phase, growth curves were obtained for each considered strain individually. For this, each strain was streaked from our glycerol stock collection onto a YPD agar plate and grown for 3 days at 30°C. Single colonies were cultivated in 15 ml YPD medium in an orbital shaker (30°C, 200 r/min, overnight). Then, each sample was diluted to an optical density (OD) at 600 nm of 0.2 in 50 ml of YPD and grown for 3 h in the same conditions (30°C, 200 r/min). Then, dilutions were made again to reach an OD of 0.1 in 50 ml of YPD in order to start all experiments with approximately the same amount of cells. The increasing growth was investigated in parallel with manual measurement of the OD from the 50-ml samples and in 100-μl samples by a microplate reader (TECAN Infinite M200). For manual measurements, we inspected the absorbance in 1 ml every 1 h. For automated measurements, the samples were centrifuged for 2 min at 3000 × *g*, washed with 1 ml of sterile water, and centrifuged again for 2 min at 3000 × *g*. The pellet was resuspended in 1 ml of sterile water. Finally, 5 μl of each sample was inoculated in 95 μl of YPD in a 96-well plate. All experiments were run in triplicate. Cultures were grown in 96-well plates at 30°C for 24 h and monitored to determine the OD every 10 min with the microplate reader. Both manual and automated OD readouts showed similar growth patterns.

Once the mid-exponential phase was determined at around 5 h for all three species, the above-mentioned protocol was repeated until all samples were growing at the exponential phase and then the cultures were centrifuged at a maximum speed of 16,000 × *g* to harvest 3 × 10^8^ cells per sample. For the cold shock experiments, when samples reached the mid-exponential phase, they were grown for 5 h more at 12°C and then the cells were harvested as described above. Total RNA from all samples was extracted using the RiboPure RNA Yeast Purification Kit (ThermoFisher Scientific) according to the manufacturer’s instructions. Total RNA integrity and the quantity of the samples were assessed using the Agilent 2100 Bioanalyzer with the RNA 6000 Nano LabChip Kit (Agilent) and NanoDrop 1000 Spectrophotometer (Thermo Scientific).

### RNA-Seq Library Preparation and Sequencing

Libraries were prepared using the TruSeq Stranded mRNA Sample Prep Kit v2 (ref. RS-122-2101/2, Illumina) according to the manufacturer’s protocol. All reagents subsequently mentioned are from this kit, unless specified otherwise. Of the total RNA, 1 μg was used for poly(A)-mRNA selection using streptavidin-coated magnetic beads. Subsequently, the samples were fragmented to approximately 300 bp. Complementary DNA (cDNA) was synthesized using reverse transcriptase (SuperScript II, Invitrogen) and random primers. The second strand of the cDNA incorporated dUTP in place of dTTP. Double-stranded DNA (dsDNA) was further used for library preparation. dsDNA was subjected to A-tailing and ligation of the barcoded Truseq adapters. All purification steps were performed using AMPure XP Beads (Agencourt). Library amplification was performed by PCR on the size-selected fragments using the primer cocktail supplied in the kit. Final libraries were analyzed using Agilent DNA 1000 chip (Agilent) to estimate the quantity and check fragment size distribution, which were then quantified by qPCR using the KAPA Library Quantification Kit (KapaBiosystems) prior to amplification with Illumina’s cBot. Libraries were loaded and sequenced 2 × 50 or 2 × 75 on Illumina’s HiSeq 2500.

### Genome-Wide Chromatin Accessibility Profiling by ATAC-Seq

The two studied strains, namely, SC and the hybrid, were grown to the mid-exponential phase in YPD at 30°C, as described above, and subjected to an assay for transposase-accessible chromatin with high-throughput sequencing (ATAC-Seq). This procedure was performed as described in Buenrostro et al. (2015) and Schep et al. (2015), with slight modifications.

For the cell nuclei preparation, approximately five million cells (counted with a hemocytometer) were harvested (centrifugation at 500 × *g* for 5 min, 4°C) and washed twice (centrifugations at 500 × *g* for 5 min, 4°C) with 50 μl of cold sorbitol buffer (1.4 M sorbitol, 40 mM HEPES-KOH, pH 7.5, 0.5 mM MgCl_2_). We used Zymolyase 100-T (ZymoResearch) to remove the cell wall, and three different concentrations were tested before proceeding with the final experiments: 1, 3, and 5 μl of Zymolyase 5 U/μl. We incubated the cells with the corresponding amount of zymolyase in 50 μl of sorbitol buffer at 30°C for 30-min shaking at 300 r/min. Then, the cells were pelleted (500 × *g* for 10 min at 4°C) and washed twice with 50 μl of cold sorbitol buffer (centrifugations at 500 × *g* for 10 min, 4°C). Fresh pellets of fungal spheroplasts were brought to the Genomics Unit at the Centre for Genomic Regulation (CRG) for further transposase reactions and library preparations for ATAC-Seq. Briefly, the nuclei were resuspended in 50 μl 1 × TD buffer containing 2.5 μl transposase (Nextera, Illumina). The transposase reaction was conducted for 30 min at 37°C. Library amplification and barcoding were performed with NEBNext Q5 Hot Start HiFi PCR Master Mix (New England Biolabs) using index primers, designed according to Buenrostro et al. (2015), at a final concentration of 1.25 μM. PCR was conducted for 12–13 cycles. Library purification was performed with Agencourt AMPure XP beads (Beckman Coulter) and library size distribution was assessed using the Fragment Analyzer (AATI, Agilent) or the Bioanalyzer High-Sensitivity DNA Kit (Agilent). The use of 3 μl of Zymolyase 5 U/μl was chosen as the optimal concentration for the experiments based on the visual inspection of the obtained profiles. ATAC-Seq libraries were quantified before pooling and sequencing using the real-time library quantification kit (KAPA Biosystems). Paired-end sequencing was performed on a HiSeq 2500 (Illumina) with 50 cycles for each read.

All experiments were performed in three biological replicates. All library preparation and sequencing steps were performed at the Genomics Unit of the Centre for Genomic Regulation (CRG), Barcelona, Spain.

### Sequencing Reads Quality Control and Visualization

We used FastQC v0.11.6^1^ and Multiqc v.1.0 (Ewels et al., 2016) to perform quality control of raw sequencing data. Adapter trimming was performed by Trimmomatic v.0.36 (Bolger et al., 2014) with TruSeq3 and Nextera adapters (for RNA-Seq and ATAC-Seq, respectively) using 2:30:10 parameters and the minimum read length of 30 bp. To visualize genomic/transcriptomic alignments and coverages, we used the Integrative Genomic Viewer v.2.3.97 (IGV) (Robinson et al., 2011).

### RNA-Seq Analysis

RNA-Seq read mapping and summarization was performed using the splice-junction aware mapper STAR v.2.5.2b (Dobin et al., 2012) with default parameters. For parental species, we mapped RNA-Seq data to the corresponding reference genomes, while for the hybrid strain, we mapped raw data to the combined *S. cerevisiae × S. uvarum* reference genomes. Further, to assess the rates of reads originated from one species while mapped to another (i.e., cross-mapping, which possibly can bias the inference of the gene expression levels), we employed two approaches: (i) mapping the reads of each parental to the concatenated reference genome and then calculating the proportion of wrongly mapped reads to a different parental genome and (ii) using the tool Crossmapper v.1.1.0 (Hovhannisyan et al., 2019), which simulates the data from both parental species, maps the reads to the concatenated genome, and calculates the cross-mapping statistics. The reference genomes and genome annotations were obtained from Ensembl (release 93; Zerbino et al., 2018) and www.saccharomycessensustricto.org (Scannell et al., 2011) for SC and SU, respectively. For SU, we merged ultrascaffolds and unplaced regions in one reference and converted GFF to GTF format using gffread v.0.9.8 (Trapnell et al., 2012) utility.

One-to-one orthologs between SC and SU were retrieved from www.saccharomycessensustricto.org (Scannell et al., 2011). Differential gene expression and ASE were assessed using DESeq2 v.1.18.0 (Love et al., 2014). For between-species comparisons, we included the matrix of gene lengths to DESeq2 object to account for their differences. Additionally, for ASE analysis (within-hybrid comparison), we supplied the DESeq2 object with the matrix of gene lengths using *normalizationFactors(dds)* < *- lengths/exp(rowMeans(log(lengths)))*, allowing DESeq2 to account only for the differences in gene lengths when calculating sizeFactors and ignoring the library size since the read counts for alleles come from the same library. For a gene to be considered differentially/allele-specifically expressed, we used a threshold of | log2 fold change| (L2FC) > 1.5 and padj (adjusted *p*-value) < 0.01, unless specified otherwise.

Differentially expressed (DE) genes were used in Gene Ontology (GO) enrichment analysis as implemented in *Saccharomyces* Genome Database (Cherry et al., 2012) to find functional enrichments in biological process, molecular function, and cellular component GO categories. GO enrichment analysis for SU was done based on SC orthologous genes. To visualize the gene expression data, we utilized ggplot2 v.2_3.0.0 R library (Wickham, 2016).

### ATAC-Seq Analysis

Data generated by ATAC-Seq were mapped to the corresponding reference genomes using BWA v.0.7.17-r1188 (Li and Durbin, 2010) with the MEM algorithm.

Initial mapping showed that ∼15–18% of reads mapped to two regions of chromosome XII (450915-469179 and 489349-490611), which contain highly repetitive ribosomal RNA (rRNA) genes of SC. Thus, to remove the adverse effects in further analysis, we have masked these two regions with bedtools maskfasta v.2.27.1 (Quinlan, 2014).

PCR duplicates were marked using Picard MarkDuplicates v.2.9.2 function^2^. We used MACS2 v.2.1.1 (Zhang et al., 2008; Li and Durbin, 2010) to perform peak calling and the bedtools genomecov to generate bedgraph files of genome coverage by ATAC-Seq reads.

Bioconductor package DiffBind v.2.4.8 (Ross-Innes et al., 2012) was used to perform general quality control and occupancy and affinity analysis of the ATAC-Seq peaks. By occupancy analysis, DiffBind finds the overall peak set between replicates of a given biological condition and/or identifies the consensus peaks between different biological conditions (i.e., parental peak set and peak set of the hybrid), while in affinity analysis it performs differential accessibility analysis of corresponding peaks, which is based on the DESeq2 workflow.

For comparing the peak sets between parentals and the corresponding homeologous chromosomes in the hybrid, we split the bam files of the hybrid into separate files for the SC and SU chromosomes using SAMtools v.1.3.1 (Li et al., 2009).

To perform differential accessibility (affinity) analysis within the hybrid, we first defined the orthologous/homeologous promoter regions as upstream, non-coding, and genomic regions up to 1 kb of length at each one-to-one orthologous locus. Defined promoter regions were usually shorter than 1 kb since the neighboring genes or chromosome borders were often encountered within that distance. We obtained bed files of the promoter regions for each species using custom python scripts. Based on the bed files, we quantified the overlapping ATAC-Seq reads within the promoter regions using bedtools multicov function. Further, differential accessibility analysis was performed using DESeq2 controlling for the length of regions.

We used bedtools closest and custom python scripts to define, for each peak, the closest upstream and downstream genes within 1-kb distance and for which the ATAC-Seq peak falls within the promoter region (Supplementary Figure S1).

### Transcription Factor Footprinting

Besides defining open chromatin regions, we used ATAC-Seq data to perform transcription factor (TF) footprinting in order to identify potential differences in TF binding site occupancy between the parental and the hybrid. Position weight matrices for the available *S. cerevisiae* TFs (*n* = 176) were retrieved from the Jaspar database (Khan et al., 2018). Footprinting was performed using the HINT software of Regulatory Genomics Toolbox v.0.11.4 (RGT) package (Gusmao et al., 2014; Khan et al., 2018; Li et al., 2019). Fungal organisms were added to HINT following the recommendations of the package developers. The *Motifanalysis* function of the RGT package was used to match the motifs of fungal TFs with the ATAC-Seq footprints. We used the *differential* function of HINT to carry out differential TF binding site occupancy analyses and generate footprinting plots. The potential targets of differentially active TFs were identified using Yeastract platform (Teixeira et al., 2018) by setting the Regulation filters to “DNA binding and expression evidence” to account only for target genes with strong experimental evidence.

All custom scripts used in this study are available at https://github.com/Gabaldonlab/Hybrid_project. Raw sequencing data of the RNA-Seq and ATAC-Seq experiments were deposited in the Sequence Read Archive under the accession numbers SRR10246851-SRR10246868 and SRR10261591-SRR10261596, respectively.

## Results

### Limited Transcriptional Impact of Hybridization

**FIGURE 1:**
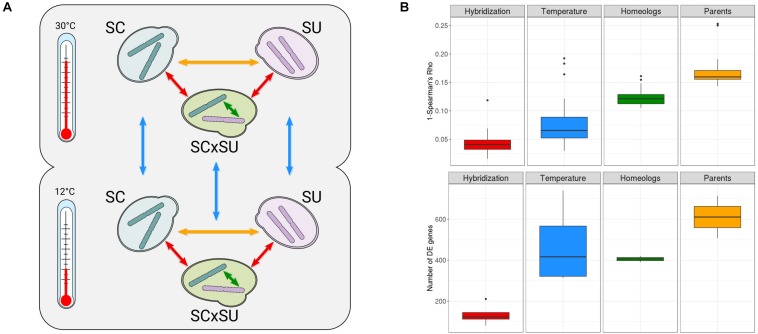
**(A)** Experimental design of the study (see section “Materials and Methods”). *Arrows* indicate comparisons of the expression levels enabled by this design: across parentals (*yellow*), genetic backgrounds (*red*), homeologous genes (*green*), and temperature conditions (*blue*). **(B)** Overall transcriptomic changes assessed as 1-Spearman’s rho correlation (*top row*) and the number of differentially expressed (DE) genes (*bottom row*). “*Hybridization*”—comparisons between parentals and hybrid at both temperatures; “*Temperature*”—comparisons of all species at two different temperatures; “*Homeologs*”—comparisons between homeologous genes at both temperatures; “*Parents*”—comparisons between parentals at both temperatures. *Colors* correspond to the comparisons depicted in **A**. A more detailed comparison for each of the above categories is depicted in Supplementary Figure S6.

To assess the impact of hybridization on gene expression, we used an RNA-Seq approach to profile the transcriptomes of diploid strains of *S. cerevisiae*, *S. uvarum*, and a *de novo* reconstructed diploid hybrid strain between these two species (see section “Materials and Methods”). We repeated the experiment at 30°C, a temperature within the optimal growth range of both species, and at 12°C, which represents a cold shock, particularly for the non-cryotolerant *S. cerevisiae* (Salvadó et al., 2011). This experimental design allowed us to directly compare transcriptional differences across genetic backgrounds (homozygous parentals and the hybrid), species (orthologous genes), homeologous chromosomes, and temperatures (see Figure 1A) and therefore assess the relative impact of these factors on gene expression levels.

As recommended for robust inference of the transcriptional levels (Liu et al., 2014), we performed all experiments in three biological replicates and sequenced over 30 million reads per replicate (see section “Materials and Methods”). The negligible level of cross-mapping between reads of the two species, as assessed by Crossmapper (Hovhannisyan et al., 2019) (Supplementary File S1), and the independent mapping of parental RNA-Seq reads to both reference genomes (Supplementary Table S1B) allowed us to accurately assign reads to each parental sub-genome in the hybrid and, thus, infer the relative expression of each of the two homeologous alleles. To additionally test whether these negligible cross-mapping rates can influence downstream results, we compared the read counts obtained from mapping parental data to the combined reference and the counts obtained by mapping parental data to corresponding parental genomes. In the case of both species, we observed Spearman’s correlations > 0.99 and that differential expression analysis with relaxed filters (| L2FC| > 1, padj > 0.05) did not show any gene affected by cross-mapping, verifying the accuracy of read assignments to corresponding species. Mapping statistics are shown in Supplementary Table S1, and quality control and reproducibility metrics for all samples are available in Supplementary Figures S2–S4. Overall, we observed lower mapping rates of SU as compared to SC, which likely reflect the lower quality of the reference assembly for the former species. For each of the 11 pairwise comparisons depicted in Figure 1A, we performed differential expression analyses (Supplementary Tables S2–S13), tested for enrichment of functional GO terms among the DE genes (Supplementary Tables S2–S13), and assessed the correlation between the levels of expression (Figure 1B).

**FIGURE 2:**
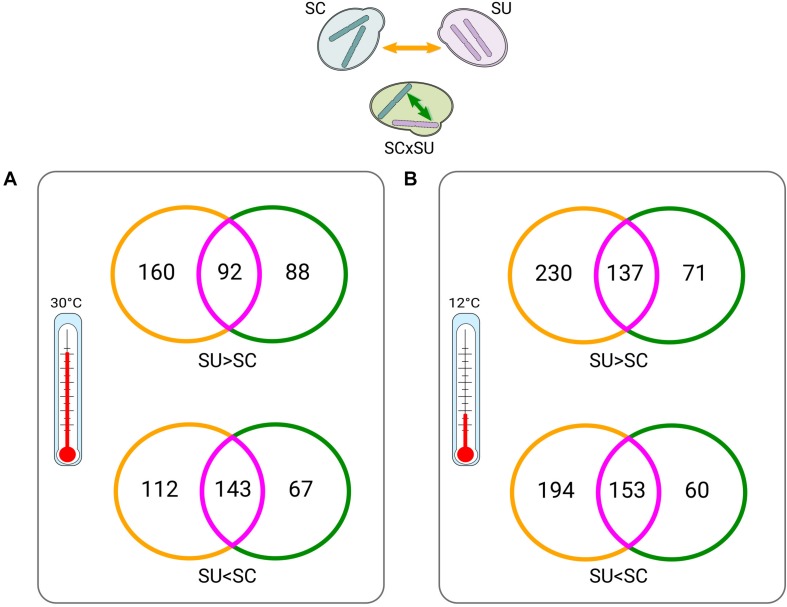
Venn diagrams of between-parent (*yellow*) *versus* within-hybrid (*green*) comparisons (depicted on the *top*) at 30°C **(A)** and at 12°C **(B)**. Intersections (*violet*) indicate differentially expressed (DE) genes that appear in both conditions. *Numbers* indicate DE genes. *Colors* of the Venn diagrams correspond to the types of comparisons, as indicated by the *arrows* in the top scheme and consistent with Figure 1A (except for intersections). “>” and “<” symbols denote which homeologs or orthologs of a given species are expressed at significantly higher and lower levels, respectively (see Supplementary Tables 4, 5, 8, 9 for lists of the DE genes).

Although not the focus of our research, we found that the cryotolerant *S. uvarum* species had a more significant rewiring of its transcriptional landscape than did *S. cerevisiae*, especially upon exposure to the lower temperature, likely reflecting an adaptive response. The observed functional enrichments among the DE genes upon change in temperature were largely consistent with previous analyses on *S. cerevisiae* and *S. uvarum* (Supplementary Tables S10, S11), such as the upregulation of chaperone activity and trehalose catabolism in low temperatures for *S. uvarum* (Li and Fay, 2017). Upregulation of trehalose metabolism in the *S. uvarum* sub-genome was also observed in the hybrid when it was exposed to 12°C (Supplementary Table S12), which might be associated with the adaptation of the hybrid to low temperatures (Pérez-Torrado et al., 2015). Most importantly, a comparison of the relative level of transcriptional differences across genetic backgrounds, temperatures, homeologous genomes, and species (Figure 2B) shows that hybridization has a rather reduced transcriptional impact, being significantly lower than that observed for the temperature shift.

Overall, only 81 and 123 genes are DE when comparing the hybrid and parental genetic backgrounds for *S. cerevisiae* and *S. uvarum*, respectively (Supplementary Tables S2, S3) at 30°C, which represents between 1 and 2% of the total gene repertoire of each species. In comparison, a temperature shift significantly alters the expression of 509 (7.1%) and 739 (11.5%) genes in these species, respectively. Additionally, we observed that the differences in expression between orthologous genes in the two species were significantly larger than those observed between homeologous genes in the hybrid. This indicates that interspecies differences in terms of transcriptional landscape are attenuated rather than increased in the hybrid.

### Low Levels of Allelic Imbalance in Yeast Hybrid

We next explored ASE in the hybrid. Consistent with the largely conserved transcriptional landscape after hybridization, we found relatively low levels of allelic imbalance (i.e., significantly different expression levels of the two homeologous genes) (Figure 1B). Specifically, at 30°C, 390 (∼7.4% of the homeologous pairs; Supplementary Table S5), homeologous genes in the hybrid show allelic imbalance, from which 180 genes show higher expression of the SU allele while 210 show higher expression of the SC allele. Thus, there is no strong preference for the hybrid to express one of the two sub-genomes. To identify whether the genes with allelic imbalance in the hybrid were a consequence of hybridization or were already DE when comparing the parental species (ASE inheritance), we compared the list of DE genes between parental species (Supplementary Table S4) with the list of imbalanced homeologous genes (Figure 2).

At 30°C (Figure 2A), 68% (143/210) and 51% (92/180) of the genes preferentially expressing the SC or SU alleles, respectively, were also found to have differential expression (with the same direction) in comparisons across species. Hence, this result indicates that the majority of genes with allele-specifically expressed genes in the hybrid are driven by inheritance of expression levels from parental species rather than resulting from the hybridization. Additionally, we found fewer genes that acquired ASE in the hybrid without being DE across species (88 and 67) as compared to genes that show no ASE despite being DE across the two species (160 and 112).

Overall, similar trends were found at 12°C (Figure 2B). Collectively, these observations suggest that hybridization tends to attenuate, rather than exacerbate, differences in the expression levels of parental orthologous genes. Finally, we also found that there is a small set (*n* = 40) of temperature-dependent allele-specifically expressed genes (Supplementary Table S13), which is congruent with an early study (Li and Fay, 2017).

### Overall Conservation of Genome-Wide Chromatin Accessibility Patterns After Hybridization

We further investigated gene regulation differences upon hybridization by performing genome-wide chromatin accessibility analysis based on ATAC-Seq at 30°C of the hybrid and the *S. cerevisiae* parental (see section “Materials and Methods” and Supplementary Table S14). We compared the ATAC-Seq profiles by performing peak calling and comparing the overlap between called peaks (i.e., inferred open chromatin regions) in the parental and the SC sub-genome of the hybrid (Supplementary Figure S5). After removing one outlier (see section “Materials and Methods”), we found that replicate experiments showed a large overlap of the called peaks (83% for both parent and hybrid replicates). Then, we compared peak sets of the SC parental with the corresponding sub-genome in the hybrid.

**FIGURE 3:**
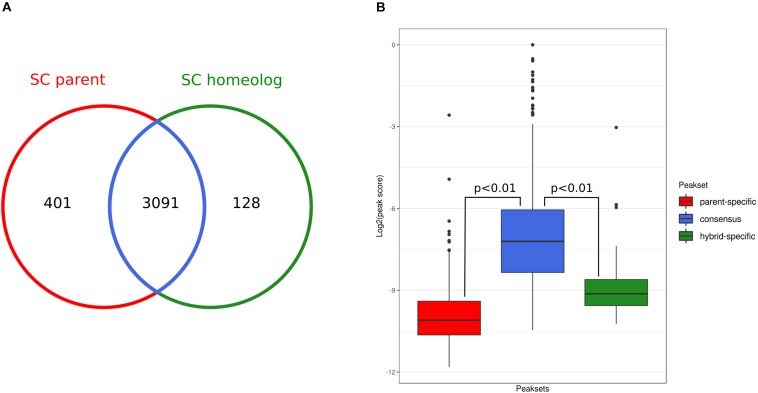
Occupancy analysis. **(A)** Overlap between ATAC-Seq peak sets detected for the *Saccharomyces cerevisiae* genome and the corresponding *S. cerevisiae* sub-genome in the hybrid. **(B)** Comparison of the distributions of DiffBind peak scores of the parent-specific (*red*), consensus (*blue*), and hybrid-specific (*green*) peaks. *P*-values are calculated using Wilcoxon test.

This analysis showed that the state of chromatin accessibility is largely similar between the SC parental genome and the corresponding sub-genome of the hybrid (Figure 3A). From 3492 parental open chromatin regions, 3,091 (88%, consensus peak set) are present in the hybrid, and conversely, 96% of the hybrid SC sub-genome peaks are shared with the SC parental. Although they represent a small fraction, we did observe parent-specific (*n* = 401) and hybrid-specific (*n* = 128) accessible chromatin regions. However, we found that these specific peaks have significantly lower scores than did the shared peaks (Figure 3B), suggesting that some of these differences might represent false-positive peak calls.

Nevertheless, to assess whether parent- and hybrid-specific peaks in chromatin accessibility were driving the observed transcriptional changes, we integrated the ATAC-Seq and RNA-Seq data. For each parental- and hybrid-specific ATAC-Seq peak, we identified the nearest downstream gene for each strand, which potentially could be regulated by the open chromatin region identified by the peak. Only one gene near a parental-specific peak was found to be also overexpressed with respect to the hybrid context: the gene encoding pyridoxal-5′-phosphate synthase (YFL059W). Conversely, the gene coding for the NADH-dependent aldehyde reductase (YKL071W) was overexpressed in the hybrid and was sitting downstream of a hybrid-specific chromatin accessibility peak. The low fraction of peaks that are specific to each genetic background, their low scores, and the very low number of downstream genes that actually show differential expression suggest that changes in chromatin accessibility upon hybridization have a very limited impact at the transcriptional level.

**FIGURE 4:**
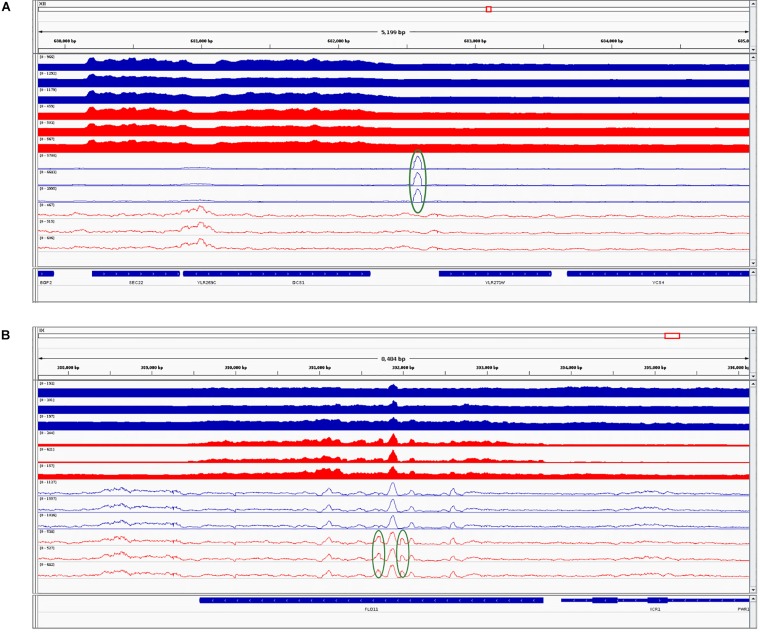
Integrative Genomic Viewer screenshots combining RNA sequencing (RNA-Seq) and assay for transposase-accessible chromatin using sequencing (ATAC-Seq) datasets for *Saccharomyces cerevisiae* (SC) and its counterpart in the hybrid. **(A)** The first identified differentially accessible region. **(B)** The second identified differentially accessible region. *Blue tracks* correspond to SC in the parental background and *red tracks* correspond to SC in the hybrid background. *Filled tracks* correspond to RNA-Seq data and *contour tracks* correspond to ATAC-Seq data. Regions with higher coverage are highlighted in *green circles*. The last track represents genomic features. Further description is given in the main text.

Further, we performed differential chromatin accessibility analysis (i.e., affinity analysis) within the consensus peak set, shared between the parental and the hybrid. Even when using liberal thresholds [L2FC > 1 and false discovery rate (FDR) < 0.01], we found only two differentially accessible peaks in this consensus set: namely, regions at chromosomes XII 682106-682709 (more open in the SC parent, L2FC = 1.3) and IX 391660-392121 (more open in the hybrid, L2FC = 1.32). We combined this result with RNA-Seq and visualized the integrated data (Figure 4).

In the first region (Figure 4A), the gene *YLR271W* that is downstream of the peak is not DE between the parent and hybrid. In contrast, the second peak (Figure 4B) coincides with the significantly higher expressed gene *FLO11* (YIR019C; L2FC = 3.44, padj < 0.01) in the hybrid as compared to the parent. However, in this case, the peak entirely overlaps the gene, and therefore it is unlikely that it regulates its expression. Thus, differential levels of chromatin accessibility do not seem to drive the few differences observed between hybrids and parental genetic backgrounds.

**FIGURE 5:**
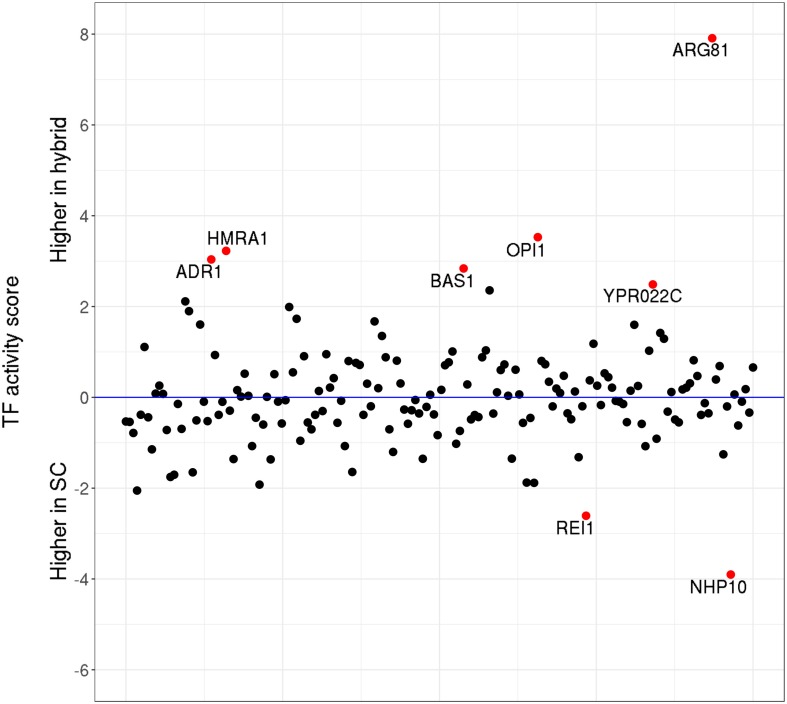
Transcription factor (TF) activity scores. Relative activity levels between *Saccharomyces cerevisiae* parental and the hybrid counterparts. *Red dots* highlight the TFs which significantly (*p* < 0.05) changed their activity levels upon hybridization.

Next, taking advantage of the high sequencing depth of our ATAC-Seq data, we also assessed the changes in TF activity (as defined by Li et al., 2019) in a genome-wide manner. The results show (Figure 5) that only eight out of 176 *S. cerevisiae* TFs have significantly (*p* < 0.05) changed their activity levels upon hybridization. In most cases, the differences in activity are moderate, with the notable exception of ARG81, which mediates the arginine-dependent repression of arginine biosynthesis genes and the activation of arginine catabolic genes.

We further identified the potential target genes for each of the deregulated TFs (Supplementary Table S15) and compared the target genes with the DE genes upon hybridization. From the 189 target genes of these eight TFs, only two genes corresponded to the genes DE in the same direction as their TF—*YHL040C* (gene encoding for siderochrome iron transporter) and *YLR346C* (encodes for a protein of unknown function), were upregulated in the hybrid genetic background. Both of these genes are regulated by BAS1, a TF involved in regulating the expression of genes of the purine and histidine biosynthesis pathways. Overall, our results suggest that, upon hybridization, the changes in TF activities are subtle and largely do not correlate with the patterns of differential gene expression observed by RNA-Seq.

### Chromatin Accessibility Patterns Within the Hybrid Are Weakly Correlated With Allele-Specific Gene Expression

Finally, we compared the chromatin accessibility profiles of the SC and SU homeologous regions within the hybrid. First, we defined the homeologous regions between two sub-genomes as maximum of 1 kb upstream regions of each one-to-one orthologous gene. Further, we quantified the ATAC-Seq coverage for these regions and performed differential accessibility analysis using DESeq2, controlling for the length differences of the regions. This analysis identified 59 and 75 genomic regions that were significantly more open or less open, respectively, in the SU sub-genome as compared to the SC sub-genome (| L2FC| > 1, padj < 0.01). By comparing these data with the results of ASE, we found that eight out of 180 preferentially expressed SU homeologs coincided with the significantly more open SU regions and that nine of 210 preferentially lower expressed SU homeologs corresponded to the significantly less open SU regions. These results are in agreement with a previously published study which identified a low correlation between changes in nucleosome positioning and gene expression levels in yeasts (Tirosh et al., 2010).

## Discussion

Fungi, and in particular Saccharomycotina yeasts, have been shown to be prone to hybridization, with an increasing number of hybrid species that are highly successful in certain niches and have industrial or clinical relevance (Pryszcz et al., 2014; Krogerus et al., 2017; Mixão et al., 2019). Hybridization has also been shown to be at the root of entire clades, e.g., the post-whole-genome duplication clade comprising *Saccharomyces* and related genera has been shown to result from a hybridization event (Marcet-Houben and Gabaldón, 2015). Thus, rather than representing evolutionary dead-ends, fungal hybrids might be highly successful and long-lived. This implies that fungal species which form a hybrid organism must overcome molecular differences and potential incompatibilities which evolved through the evolutionary history of the parentals. On the relatively well-studied genomic level, fungal hybrids, and in particular those from the *Saccharomyces* genus, tend to undergo genomic rearrangements, including loss of heterozygosity, gene conversion, and partial or full chromosome loss, among others, that help to overcome incompatibilities and stabilize genomes, which results in genome mosaicism.

In our study, we assessed how the two distantly related *Saccharomyces* species cope with hybridization at the levels of the transcriptome and chromatin landscapes.

Collectively, our results show that, despite genome merging of extremely diverged species, hybridization has a comparatively smaller effect on the transcriptome than a shift from temperate to cold temperature. Moreover, we found that most loci express the two homeologous alleles in similar proportions, and those genes that show ASE largely overlap with those whose orthologs in the two parental species already show different levels of expression, suggesting that most of the ASE derives from already existing interspecies differences. Furthermore, homeologous genes within the hybrid showed fewer differences than did orthologous genes in the two parental species, indicating that, rather than being exacerbated, interspecies differences in expression are attenuated upon hybridization. This is consistent with an earlier study on a natural hybrid of the genus *Epichloë* (Cox et al., 2014). However, as our model involves a newly formed hybrid, our study clarifies that the attenuation of the differences is an immediate effect of hybridization and not the result of adaptation through evolution of the hybrid lineage. Additionally, Cox et al., proposed that with an increase in genome divergence between the parentals, which ∼ 5% in *Epichloë* (Campbell et al., 2017), the magnitude of transcriptome shock will increase accordingly, while our results demonstrate that despite a large evolutionary distance of 20% of nucleotide divergence in coding regions (Kellis et al., 2003) between SC and SU the consequences of hybridization are still buffered. Moreover, in contrast to the *Epichlöe* study that compares haploid parentals with a diploid hybrid, our study compares diploid parental strains and diploid hybrids and thus avoids any potential misleading effect resulting from a ploidy change.

The absence of a large impact of hybridization in gene expression in fungal hybrids is in stark contrast with what has been reported in animals or plant studies (McManus et al., 2010; Yoo et al., 2013; Li et al., 2014; Wu et al., 2018; Zhang et al., 2018), where widespread changes in expression following hybridization have been observed. For instance, in newly resynthesized allotetraploid *Brassica napus* (Wu et al., 2018), 30.4% of the genes showed expression changes upon hybridization compared to its diploid parentals *Brassica rapa* and *Brassica oleracea*, and over 90% of the deregulated genes were downregulated in the allotetraploid compared to the parents. Additionally, 36.5% of homeologous pairs within the hybrid *B. napus* displayed differential expression toward either of the alleles, with a slight preference to the *B. rapa* parental. Similarly, in allopolyploid cotton *Gossypium arboreum (A2) × Gossypium raimondii (D5)*, 22–30% of parental genes showed differential expression in the hybrid as compared to the parentals (Yoo et al., 2013). The study of the allelic imbalance of a synthetic hexaploid wheat showed that 24.1% of the identified homeologous genes were imbalanced in the hybrid, and this difference in expression could not be attributed to preexisting expression divergence between the parentals (Li et al., 2014). Finally, a recent study on the diatom microalgae *Fistulifera solaris* showed that ∼61% of homeologous genes displayed allelic imbalance (Nomaguchi et al., 2018).

For *Drosophila* hybrids, different amplitudes of transcriptome misexpression have been previously reported, which depended on the level of genetic divergence of the parental species. For instance, a recent transcriptomic study of *Drosophila mojavensis* and *Drosophila arizonae* (diverged 0.6–1 mya) and their hybrid showed that 12% of genes in the hybrid are DE as compared to the parents (Lopez-Maestre et al., 2017). This is much larger than the fraction of DE genes in this study despite the much lower genetic distance in the *Drosophila* species. The same study showed that 8% of genes between parentals have diverged expression. That is, in that case, differential expression between homeologous genes in the hybrid was more abundant than between orthologous genes in the two parental species, which is the contrary of what we have observed for the *Saccharomyces* hybrid in this study. On the other hand, the comparison between more diverged fly species, namely, *Drosophila melanogaster* and *Drosophila sechellia* (diverged ∼1.2 mya), identified 78% of DE genes between parents (McManus et al., 2010). Interestingly, a recent study of hybrid chicken breeds (intraspecies hybrids) showed tissue-dependent rates of gene expression divergence: while it was ∼15% of genes in the liver, gene expression divergence between parental breeds in the brain was as low as 0.8% of genes (Gu et al., 2019).

It must be noted that comparing results across species and studies is difficult. These studies were performed using different technologies and data analysis methods, which makes direct comparisons problematic. For instance, Wu et al. (2018) used fragments per kilobase of transcript per million mapped reads (FPKM) expression values and applied a filter of FDR ≤ 0.05 and absolute log2 fold change ≥ 1 for a gene to be considered as DE. Yoo et al. (2013) used raw read counts for differential expression analysis and a filter of adjusted *p*-value < 0.05 with no filtering on fold change. Gu et al. (2019) applied an absolute fold change ≥ 1.25 and FDR < 0.5 for assigning DE genes in chicken hybrid breeds, and Lopez-Maestre et al. (2017) used FDR < 0.01 and log2 fold change > 1.5 for assigning DE genes in *Drosophila*, which are the filters also used in our study. In order to assess how data filtering can influence our results, we applied a set of more liberal filters for finding DE genes upon parental–hybrid transition. With padj (FDR) < 0.05, | log2 fold change| > 1, and mean normalized expression levels > 10 (which, in fact, take into account only the expressed part of the transcriptome, limiting transcriptome-wide inferences), we obtained, on average, ∼4.6 and ∼9% of DE genes for SC and SU, respectively, across temperatures. This shows that, even with relaxed filters, transcriptome shock in our yeast hybrid is lower than in plants and animals. Thus, methodological differences notwithstanding, the different animal and plant studies seem to agree in reporting a large transcriptomic impact of hybridization, as well as large levels of allele-specific imbalance, whereas the fungal studies consistently report more moderate effects.

We further assessed the impact of hybridization on another level: that of chromatin accessibility. Here, consistent with the low level of differences in gene expression, we found minor differences in terms of chromatin accessibility and TF activity between the hybrid and the *S. cerevisiae* parental. Admittedly, subtle differences in TF expression might have significant biological effects. However, our results suggest that the few observed differences in chromatin accessibility and TF activity are not driving the few observed differences in gene expression levels.

Based on our results and those from other previous studies, we hypothesize that unicellular fungi and multicellular plants and metazoans respond fundamentally differently to hybridization in terms of transcriptional response, which may explain why hybridization is so common in fungi, and, as compared to plants and animals, it can encompass larger genetic distances (Morales and Dujon, 2012). What molecular phenomena govern these different responses to hybridization? One could argue that differences in the magnitudes of transcriptomic shock in fungi and metazoans and plants can be attributed to differences in the mechanisms regulating gene expression. Though the general and fundamental principles of transcriptional regulation are largely conserved across eukaryotes, the complexity of gene regulation in plants and animals is more sophisticated than that in fungi (Rando and Chang, 2009; Hahn and Young, 2011; Lelli et al., 2012). For example, plants and animals possess a richer repertoire of chromatin modification regulators as compared to yeasts, which provide them with additional layers of regulation and a more sophisticated fine-tuning of the expression levels (Rando and Chang, 2009).

Additionally, fungi and yeasts in particular are prone to genomic rearrangements, ranging from small indels to large-scale copy number variations, inversions, translocations, and duplications (Albertin and Marullo, 2012; Plissonneau et al., 2016; Möller and Stukenbrock, 2017; Möller et al., 2018; Steenwyk and Rokas, 2018). Not only are these genomic alterations compatible with fungal viability but also, inversely, can promote fitness and adaptability to different niches (Selmecki et al., 2005, 2009; Croll et al., 2013; de Jonge et al., 2013; Gabaldón and Carreté, 2016; Mixão and Gabaldón, 2018). Hence, one could expect that, following hybridization, two complex gene regulatory systems, such as that of animals and plants, are more likely to experience larger levels of incompatibilities and perturbations as compared to simpler and more versatile regulatory systems, such as those of yeasts.

In this context, Cox et al. (2014) introduced the concept of “modulon” which encompasses all gene regulatory mechanisms, including *cis-* and *trans-*regulation, posttranscriptional regulation, TFs, epigenetics, etc. Differences in the levels of expression of orthologous genes in different species arise due to differences in the species’ modulons, which have evolved independently for some time. Upon hybridization, several regulatory scenarios can take place: (i) modulons of the two species have no or little crosstalk with each other because they are too divergent; (ii) modulons are largely similar and compatible with each other, resulting in a so-called homeolog expression blending; or (iii) modulons of the two species preferentially target one of the alleles. Importantly, these regulatory outcomes can coexist, affecting different portions of the transcriptome, which can be quantitatively assessed. In the first scenario, the genes from the parental species would inherit their expression levels in the hybrid with no subsequent expression alterations. This outcome accounts for the majority of genes in our study: ∼92 and ∼89.3% of orthologous genes at 30 and 12°C, respectively (non-DE orthologs + violet parts in Figure 2). The second scenario will result in diminished differences in homeologous expression levels as compared to differences across species. In our study, this could account for ∼5.24 and ∼8.2% of orthologous genes at 30 and 12°C, respectively (yellow parts in Figure 2). In the third case, homeologous genes will acquire divergence in gene expression that was not observed in parentals, which represents the transcriptomic shock caused by hybridization. In our study, this accounts for ∼2.9 and ∼2.52% of homeologous genes at 30 and 12°C, respectively (green parts in Figure 2).

Altogether, our study suggests a conservative and restricted impact of hybridization at the transcriptomic and chromatin profiles in hybrid yeast, which can be largely attributed to the absence of a regulatory crosstalk between highly diverged fungal modulons. We hypothesize that the moderate impact that hybridization has on the levels of chromatin accessibility and gene expression is at the root of the strong ability for successful hybridization in yeasts and other fungi. Further research involving diverse taxonomic groups of fungi is required to address this hypothesis in order to disentangle the role of transcriptome and chromatin profile buffering in fungal hybridization.

## Data Availability Statement

Raw sequencing data of RNA-Seq and ATAC-Seq experiments were deposited in the Sequence Read Archive under the accession numbers SRR10246851-SRR10246868 and SRR10261591-SRR10261596, respectively.

## Author Contributions

TG, ES, and HH designed the study. ES and EK performed the experiments. HH analyzed the data. HH and TG interpreted the results and prepared the manuscript. AH and EL constructed the strains used in the study. All the authors have read and approved the final manuscript.

## Conflict of Interest

The authors declare that the research was conducted in the absence of any commercial or financial relationships that could be construed as a potential conflict of interest.

## Footnotes

1 http://www.bioinformatics.babraham.ac.uk/projects/fastqc

2 http://broadinstitute.github.io/picard
