# Supplementary material for: Integrative Omics Analysis Reveals a Limited Transcriptional Shock After Yeast Interspecies Hybridization
Source: Front Genet. 2020 May 7;11:404. doi: 10.3389/fgene.2020.00404 (PMC7221068; doi:10.3389/fgene.2020.00404)
Supplement: Supplementary file 16 [file Data_Sheet_1.PDF]

To view Supplementary file 1, please follow the link https://www.dropbox.com/s/ntmi000ucrepeh1/Supp_file_1.html?dl=0, download and open the file in your browser (preferably Google Chrome or Firefox).
