# Supplementary material for: Integrative Omics Analysis Reveals a Limited Transcriptional Shock After Yeast Interspecies Hybridization
Source: Front Genet. 2020 May 7;11:404. doi: 10.3389/fgene.2020.00404 (PMC7221068; doi:10.3389/fgene.2020.00404)
Supplement: Supplementary file 17 [file Image_1.PDF]

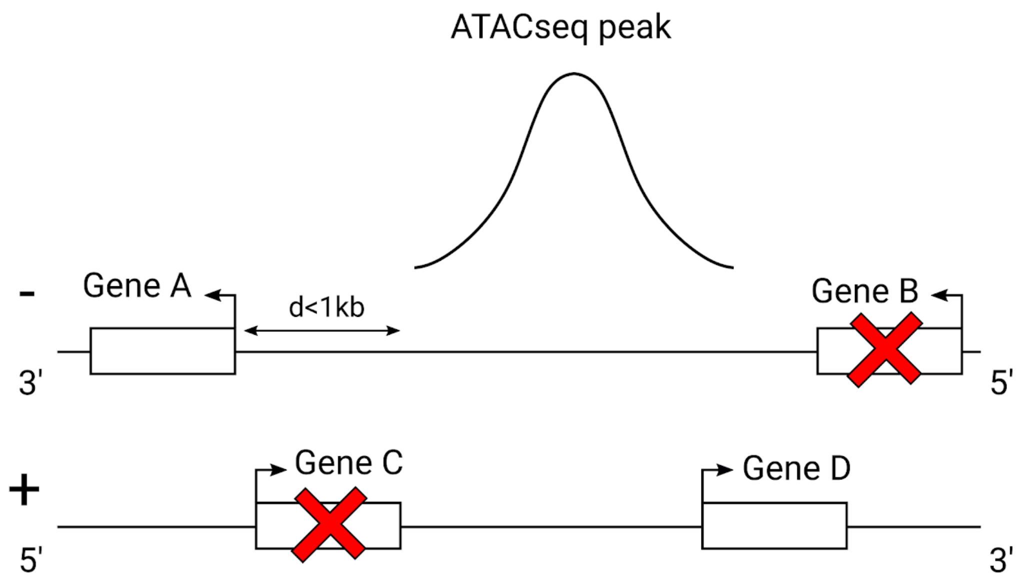

Supplementary Figure 1. Schematic representation of proximal gene selection relative to ATAC-seq peaks. Only down-stream genes (Gene and Gene D) of the peak relative to the strand (“+” and “-”) can be potentially regulated by the peak, since promoter regions of up-stream genes (Gene B and Gene C) are not influenced by the peak. “d<1kb” indicates that the distance between the peak and the genes does not exceed 1 kb.
