# Supplementary material for: Integrative Omics Analysis Reveals a Limited Transcriptional Shock After Yeast Interspecies Hybridization
Source: Front Genet. 2020 May 7;11:404. doi: 10.3389/fgene.2020.00404 (PMC7221068; doi:10.3389/fgene.2020.00404)
Supplement: Supplementary file 19 [file Image_3.PDF]

A.

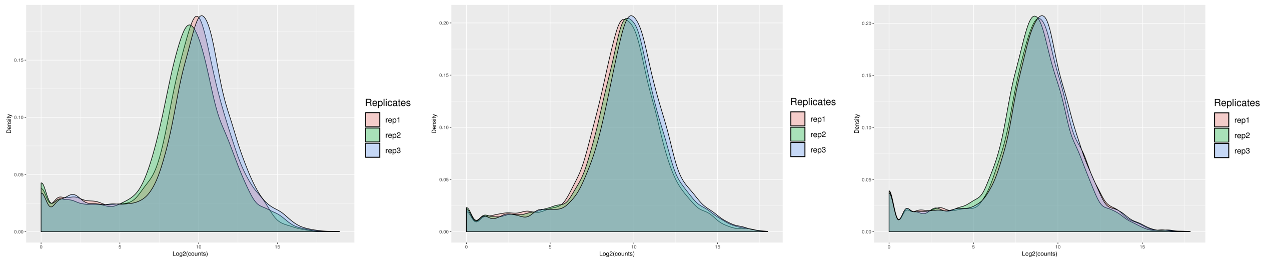

B.

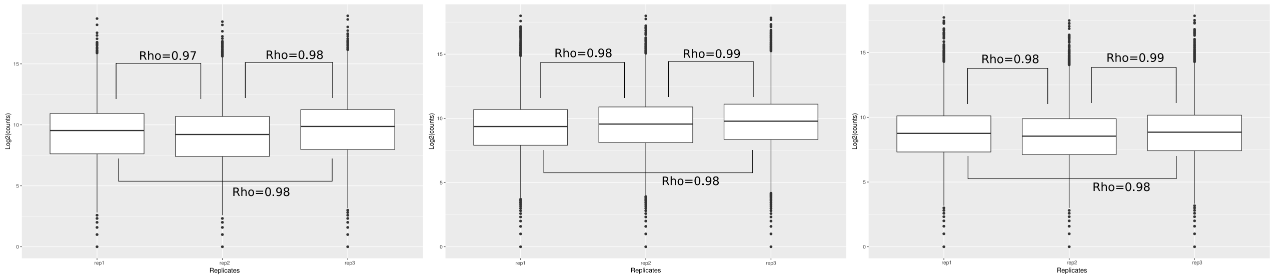

Supplementary Figure 3. Quality control and reproducibility metrics of RNA-Seq samples obtained at 12C.

A. From left to right: density plots of Log2 raw read counts for SC, SU and the hybrid, respectively.

B. From left to right: box-plots of Log2 raw read counts for SC, SU and the hybrid, respectively. “Rho” denotes Spearman’s rho correlation coefficient between pairs of biological replicates; “rep” denotes biological replicates.
