# Supplementary material for: Integrative Omics Analysis Reveals a Limited Transcriptional Shock After Yeast Interspecies Hybridization
Source: Front Genet. 2020 May 7;11:404. doi: 10.3389/fgene.2020.00404 (PMC7221068; doi:10.3389/fgene.2020.00404)
Supplement: Supplementary file 20 [file Image_4.PDF]

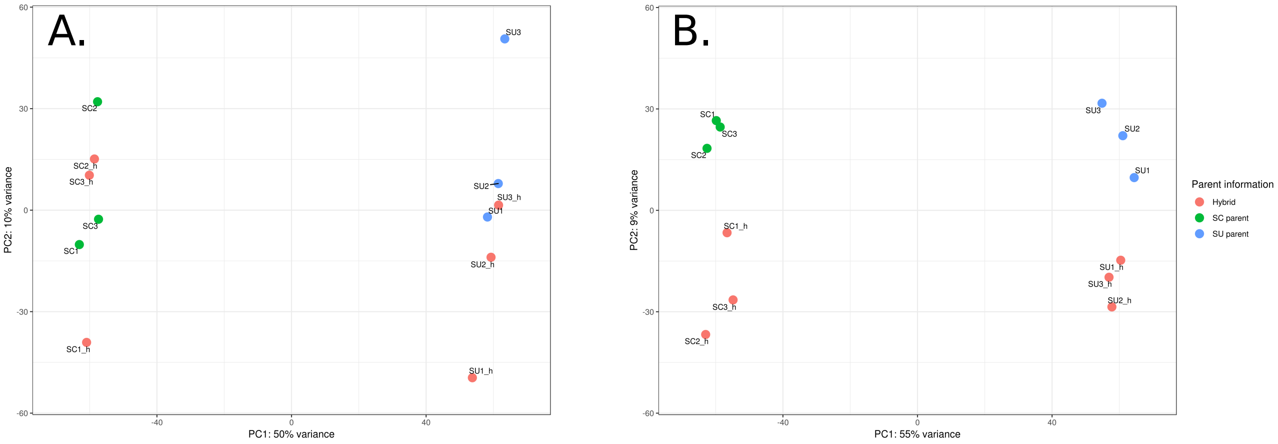

Supplementary Figure 4. Principal Component Analysis plots for studies samples. **A.** Parental and homeologous samples at 30C; **B.** Parental and homeologous samples at 12C.
