# Supplementary material for: Integrative Omics Analysis Reveals a Limited Transcriptional Shock After Yeast Interspecies Hybridization
Source: Front Genet. 2020 May 7;11:404. doi: 10.3389/fgene.2020.00404 (PMC7221068; doi:10.3389/fgene.2020.00404)
Supplement: Supplementary file 21 [file Image_5.PDF]

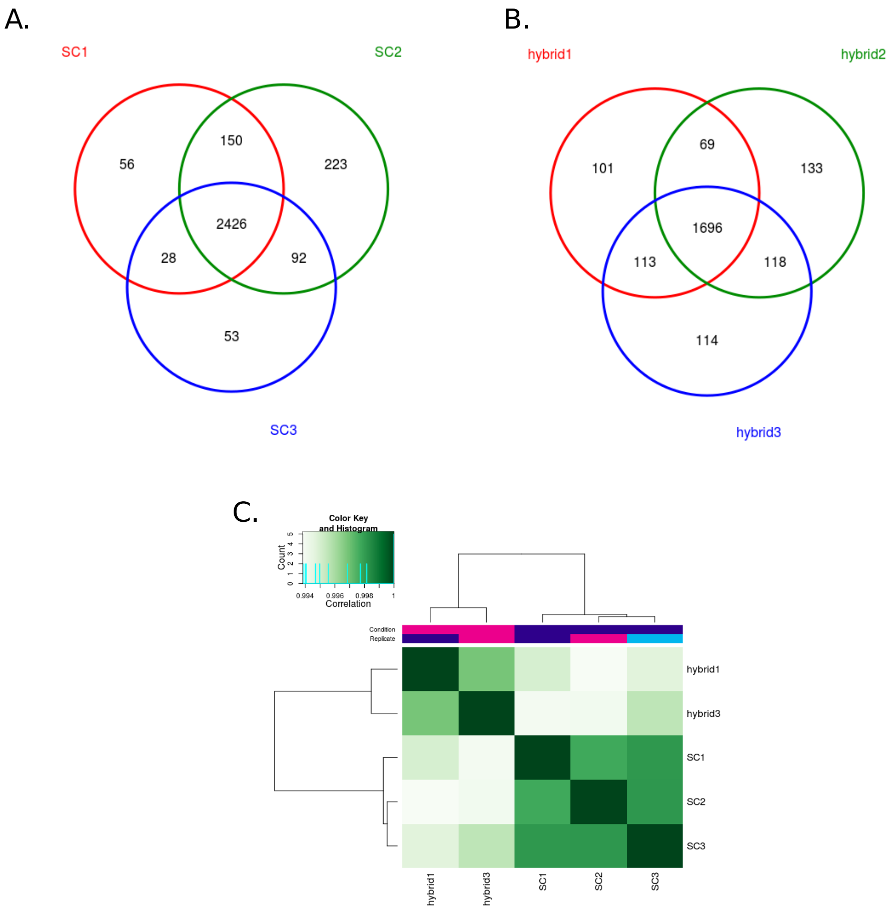

Supplementary Figure 5. Quality control plots of ATAC-Seq peaks of SC and SC homeolog. **A.** Venn diagram of peaks called in SC parental. **B.** Venn diagram of peaks called in SC homeolog in hybrid. **C.** Correlation heat map of studied samples. Sample hybrid2 is removed as it was an outlier.
