# Supplementary material for: Integrative Omics Analysis Reveals a Limited Transcriptional Shock After Yeast Interspecies Hybridization
Source: Front Genet. 2020 May 7;11:404. doi: 10.3389/fgene.2020.00404 (PMC7221068; doi:10.3389/fgene.2020.00404)
Supplement: Supplementary file 22 [file Image_6.PDF]

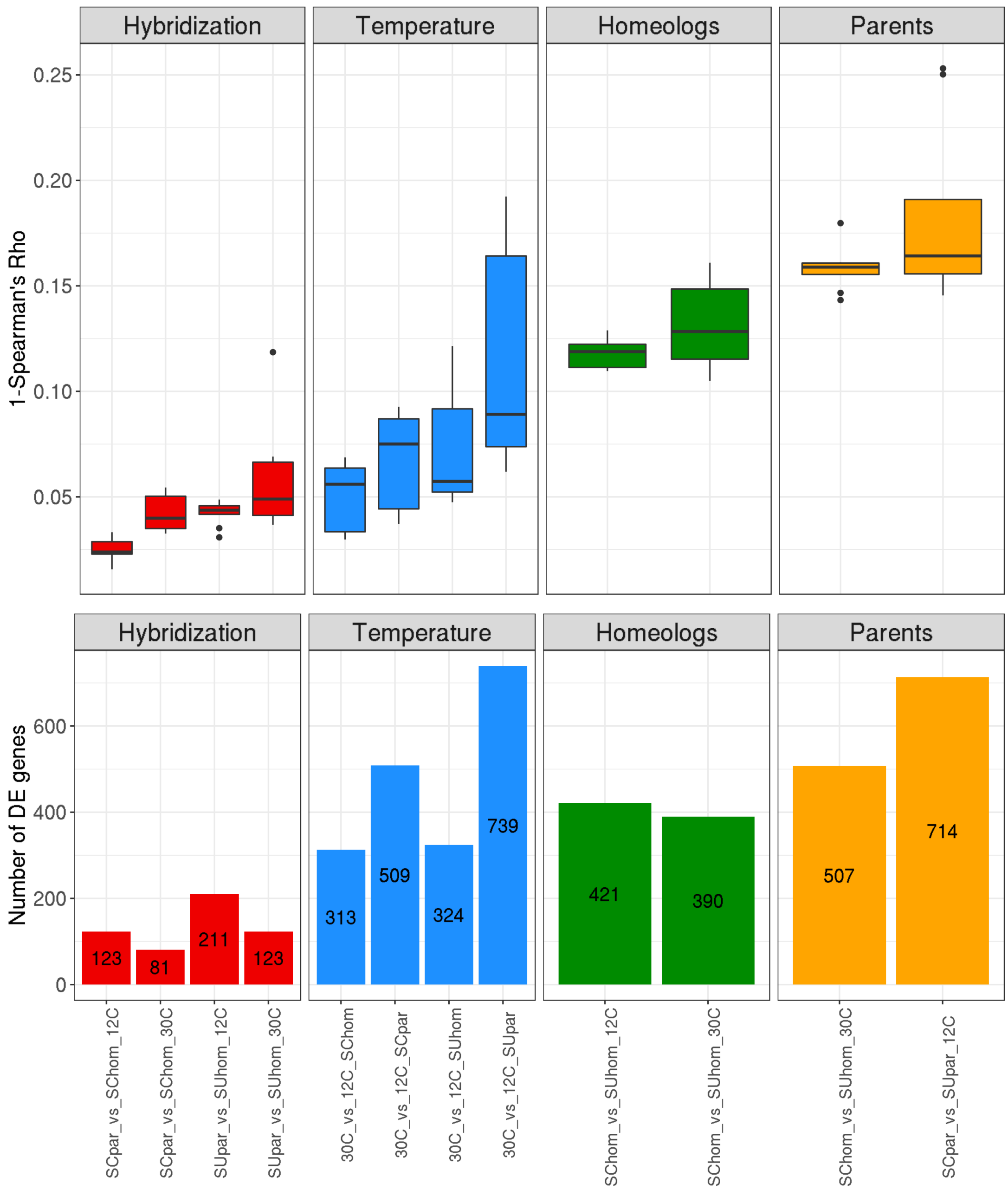

Supplementary Figure 6. Detailed overall transcriptomic changes assessed as 1-Spearman's Rho correlation (top row) and the number of DE genes (bottom row). "Hybridization" - comparisons between parentals and hybrid at both temperatures; "Temperature" - comparisons of all species at two different temperatures; "Homeologs" - comparisons between homeologous genes at both temperatures, "Parents" - comparisons between parentals at both temperatures.
